# Can Salivary Biomarkers Serve as Diagnostic and Prognostic Tools for Early Detection in Patients with Colorectal Cancer? A Systematic Review

**DOI:** 10.3390/cimb47080647

**Published:** 2025-08-12

**Authors:** Stanisław Krokosz, Maria Obrycka, Anna Zalewska

**Affiliations:** 1 Department of Restorative Dentistry, Medical University of Bialystok, 15-089 Białystok, Poland; 2 Students’ Research Group of Department of Restorative Dentistry, Medical University of Bialystok, 15-089 Białystok, Poland; 3 Independent Laboratory of Experimental Dentistry, Medical University of Bialystok, 15-089 Białystok, Poland

**Keywords:** colorectal cancer, biomarkers, metabolomics, saliva, liquid biopsy, diagnostics

## Abstract

Colorectal cancer (CRC) stands as one of the most prevalent and lethal forms of cancer worldwide with early detection playing a crucial role in improving the survival rate. Salivary biomarkers have emerged as a promising non-invasive alternative for CRC early detection. A comprehensive search of the Web of Science, Scopus, and PubMed databases was performed to identify relevant studies published between 2018 and April 2025. Inclusion criteria focused on studies analyzing salivary biomarkers in adult CRC patients, while pediatric studies, non-diagnostic applications, and studies with insufficient statistical power were excluded. A total of 12 studies were included in this review, identifying various salivary biomarkers associated with CRC. Salivary microbiota, including *Fusobacterium nucleatum* and other bacterial species, demonstrated potential as diagnostic markers. Metabolomic profiling revealed elevated levels of lactate and pyruvate, reflecting metabolic alterations in CRC. Several microRNAs, such as miR-92a and miR-29a, exhibited high sensitivity and specificity for CRC detection. Additionally, protein-based biomarkers, including chemerin and sHLA-G, were found to be significantly elevated in CRC patients. Salivary biomarkers show great promise as a non-invasive, cost-effective approach for CRC detection and prognosis. Their ability to reflect systemic disease processes makes them a valuable complement to existing screening methods.

## 1. Introduction

Cancer poses the highest clinical, social, and economic burden and is the second leading cause of death globally, accounting for an estimated 9.6 million deaths, or 1 in 6 deaths, in 2018 [1,2,3,4]. The development of cancer cell tumorigenesis in the human body is a result of many internal and external factors (carcinogens), such as genetic liability, sporadic mutations, lifestyle, substance abuse, and viral and bacterial infections [5,6,7].

Colorectal cancer (CRC) is among the most common and lethal malignancies globally, currently ranking third in incidence and second in cancer-related mortality, underscoring its substantial burden on global public health [1,8]. Colorectal cancer (CRC) arises through a multistep adenoma–carcinoma sequence initiated by the accumulation of oncogenic mutations (e.g., loss of the APC tumor suppressor gene leading to constitutive Wnt/β-catenin signaling), followed by the activation of alterations in KRAS and inactivation of TP53, which together drive aberrant crypt formation and uncontrolled proliferation in the colonic epithelium [9,10,11]. These early genetic hits give rise to benign adenomatous polyps that, if left untreated, accumulate further genomic and epigenetic changes, such as DNA mismatch repair deficiency (microsatellite instability) or CpG island methylator phenotype, promoting invasion through basement membrane degradation (via upregulation of matrix metalloproteinases) and ultimately culminating in an invasive carcinoma [12,13,14,15,16,17]. Although CRC is a disease that can be prevented or effectively treated if diagnosed early, mortality remains high due to the lack of signs and symptoms during the early cancer stages [18]. Identifying the disease in its pre-symptomatic stage substantially enhances patient survival compared with diagnoses made after symptom onset [19,20].

In the current detection and diagnosing strategies, colonoscopy has been chosen as the effective ‘gold standard’; nevertheless, it exhibits many limitations and weaknesses [21,22,23,24]. Colonoscopy, despite its diagnostic accuracy, is an invasive procedure that necessitates extensive patient preparation and skilled endoscopic expertise and often entails prolonged scheduling delays. These logistical and procedural drawbacks can defer disease detection and diminish survival outcomes, while also discouraging patient adherence to recommended screening protocols [19,20,21,22,23,24].

Computed tomography colonography and capsule endoscopy provide comprehensive, non-invasive visualizations of the colonic lumen, yet both modalities carry significant drawbacks: both do not allow for tissue biopsy or histopathological sampling and rely heavily on the quality of bowel preparation and the expertise of the interpreting clinician [25,26,27]. Moreover, CT colonography exposes patients to ionizing radiation and involves high capital expenditure for scanners and software, whereas capsule endoscopy depends on expensive, single-use devices and specialized image-analysis workstations and is still less sensitive in detecting malignancies than traditional colonoscopy [28,29,30,31,32].

Fecal immunochemical tests (FIT) and guaiac-based fecal occult blood tests (gFOBT) are screening methods that detect the presence of hidden blood in stool samples [33]. Although blood in the stool may indicate bleeding polyps or malignancies, positive fecal occult blood tests might be due to different conditions such as peptic ulcers, inflammatory bowel disease or even may be related to medication use [34,35,36,37,38,39]. Although fecal occult blood testing remains a mainstay of CRC screening, its diagnostic performance is constrained by suboptimal sensitivity and specificity, particularly in identifying non-hemorrhagic lesions and early-stage adenomatous polyps, leading to false-negative results and delayed intervention [39,40]. Common blood biomarkers for colorectal cancer, such as the carcinoembryonic antigen (CEA) and carbohydrate antigen 19-9 (CA 19-9), are mainly used to monitor treatment response and detect recurrence as they also have limited sensitivity and specificity in early-stage disease [24]. Having considered the characteristics of the mentioned diagnostic tests creates an urgent need for new, non-invasive, highly sensitive, and specific, patient-friendly diagnostic tests.

**Figure 1:**
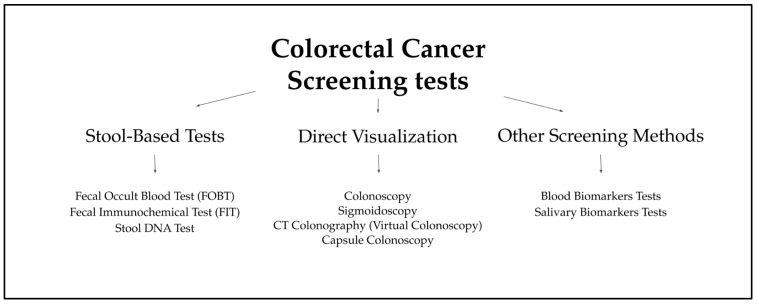
Types of CRC Screening Tests [18,19,20,21,22,23,24,25,26,27,28,29,30,31,32,33,34,35,36,37,38,39,40,41,42,43,44,45,46,47].

Cancer cells exhibit a short lifespan, releasing products of cellular degradation, such as circulating tumor cells (CTCs), cell-free circulating nucleic acids (cfDNA/RNA), microRNAs (miRNAs), long non-coding RNAs (lncRNAs), exosomes, and proteins, from primary or metastatic tumors into the extracellular environment [41,42,43,44,45]. These biomarkers are directly or indirectly secreted into bodily fluids such as peripheral blood, urine, or saliva, providing critical insights into physiological processes at the single-cell level [43,44,45]. Among modern diagnostic approaches, salivary biomarkers hold significant promise due to noninvasiveness with straightforward sample collection and processing [46,47] (Figure 1, Table 1).

Saliva is a complex fluid composed of secretions from the salivary glands and gingival crevicular fluid, the latter of which closely resembles serum in its composition [47,48]. It comprises inorganic and organic substances, including proteins, enzymes, DNA, mRNA, miRNA, antibodies, tissue metabolism products, and microorganisms [48]. Each component of saliva plays a well-defined role in maintaining the homeostasis of the oral cavity and the entire organism [48,49]. Salivary diagnostics offer several distinct advantages over other colorectal cancer detection methods [50]. Unlike endoscopic procedures, saliva collection is completely non-invasive and painless [51]. It does not require bowel preparation, radiation exposure, or clinical supervision, in contrast to CT colonography or endoscopy, making it highly acceptable to patients. It can be easily self-collected, perhaps even outside of clinical settings, which may increase screening participation and accessibility, particularly in underserved or rural populations. Given its serum-like molecular profile and accessible collection, saliva has recently emerged as a powerful biofluid for noninvasive biomarker discovery. This review critically examines the potential of salivary constituents for the early detection, prognostication, and therapeutic monitoring of one of the most prevalent cancers worldwide—colorectal carcinoma [52,53,54].

## 2. Materials and Methods

To provide an overview of the salivary biomarkers characteristics of colorectal cancer, we conducted a systematic review using three databases: Web of Science, Scopus, and PubMed. Google Scholar was excluded for its lack of transparency, limited search functionality, and inclusion of non-peer-reviewed sources. The review was made according to the guidelines of the Preferred Reporting Items for Systematic Reviews and Meta-Analyses 2020 (PRISMA 2020) framework [55,56]. The search formulas included:

1. For Web of Science: TS = ((colorectal cancer OR colon cancer OR rectal cancer) AND saliva AND (biomarkers OR markers)) and TS = ((oral OR saliva) AND (liquid biopsy))
2. For Scopus: TITLE-ABS-KEY ((colorectal cancer OR colon cancer OR rectal cancer) AND saliva AND (biomarkers OR markers)) and TITLE-ABS-KEY ((oral OR saliva) AND (liquid biopsy))
3. For PubMed: (colorectal cancer OR colon cancer OR rectal cancer) AND saliva AND (biomarkers OR markers) and (oral OR saliva) AND (liquid biopsy)

We included studies researching biomarkers found in body liquids of adult patients with colorectal cancer, including rectal and colon cancer and systematic reviews on the topic of CRC biomarkers, published between 2014 and April 2025. The first search was conducted on 12 October 2024, and the last search was conducted on 30 April 2025. Exclusions were applied to studies published in different languages than English, pediatric studies, non-diagnostic or prognostic applications, insufficient sample size or poor statistical analysis, non-clinical studies, case reports, expert opinions, and editorials.

Titles and abstracts were screened independently by two reviewers (S.K. and M.O.), with full-text articles reviewed to confirm eligibility based on inclusion and exclusion criteria. Disagreements were resolved through discussion and arbitration by a third reviewer (A.Z.). Efforts were made to prevent duplicate or overlapping data, with duplicates manually removed using Zotero software (Version: 7.0.20; Corporation for Digital Scholarship, Vienna, Virginia, USA) by one reviewer (S.K.).

The PECO variant of the PICO framework was used for the inclusion and exclusion of studies as shown in Table 2. Detailed search protocol is presented in Section 3 using the PRISMA protocol [45,46].

### Quality Assessment and Critical Appraisal for the Systematic Review of Included Studies

**Figure 2:**
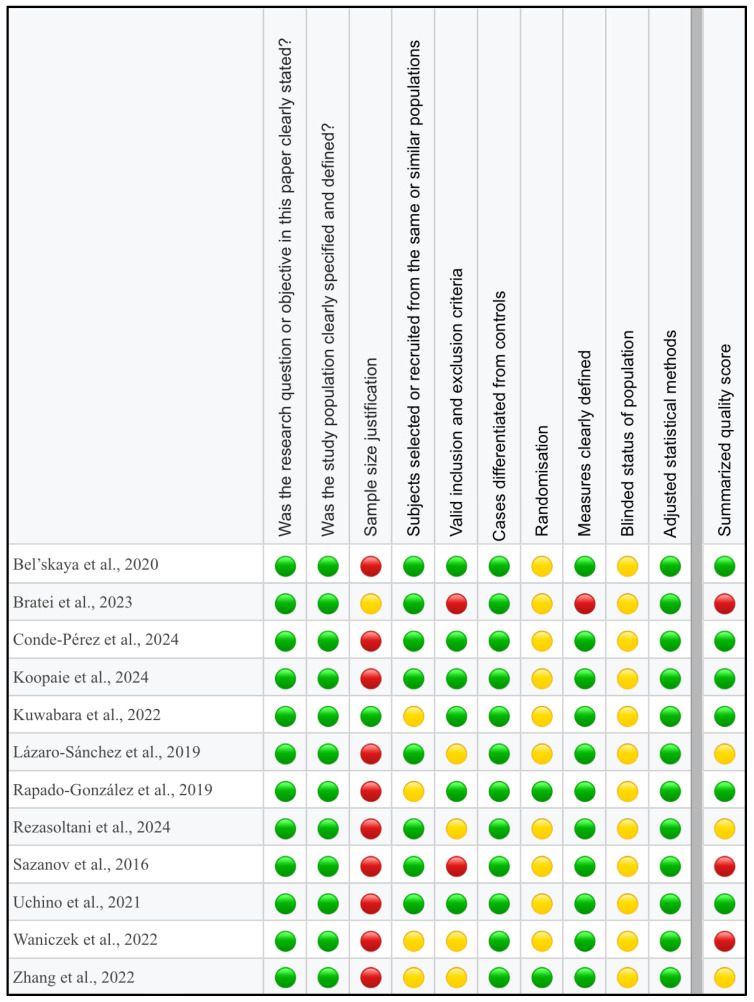
Quality assessment, including the main potential risk of bias (risk level: green—low, yellow—unspecified, red—high; quality score: green—good, yellow—intermediate, red—poor) [58,59,60,61,62,63,64,65,66,67,68,69].

The ‘Study Quality Assessment Tool’ issued by the National Heart, Lung, and Blood Institute, National Institute of Health was used to assess the risk of bias in each of the individual studies included in this review [57]. The questionnaires for every study were completed independently by two reviewers (S.K. and M.O.), with disagreements resolved through discussion and arbitration by a third reviewer (A.Z.). The summarized quality assessment for every single study is reported in Figure 2.

Oxford center for Evidence-Based Medicine Levels for Evidence was used to assess the evidence of reviewed studies; all of them showed a level three or four out of five in terms of evidence [70].

## 3. Results

**Figure 3:**
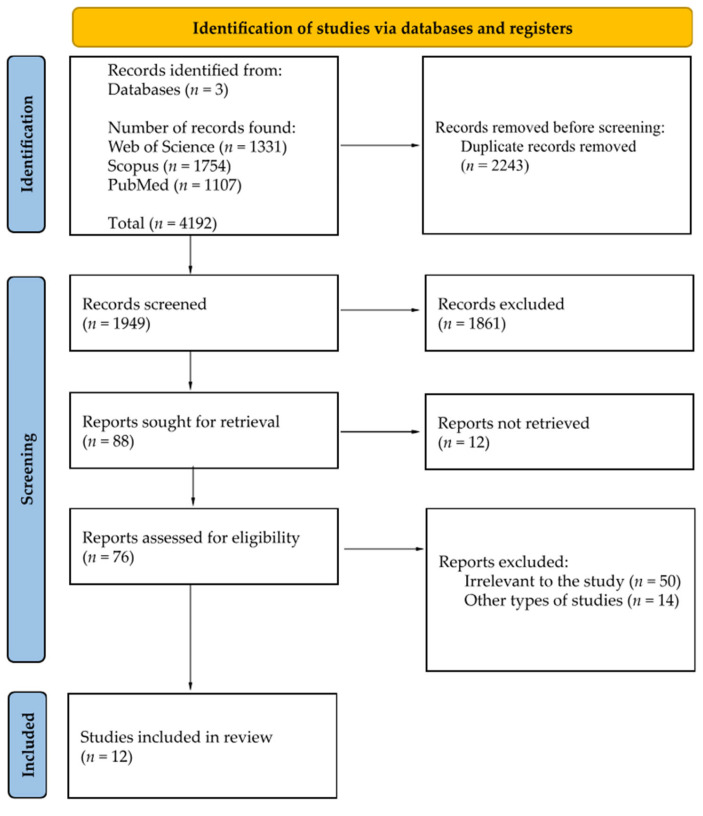
PRISMA 2020 flow diagram [55].

Out of 4192 publications, 13 were identified as meeting the specified inclusion and exclusion criteria. The literature review uncovered 4192 entries in the PubMed, Scopus and Web of Science databases, with the following records removed before screening: 2243 duplicate records removed. A total of 1949 papers were screened; 1861 records were excluded based on title; 12 reports out of 88 were not retrieved in full-text availability. Out of 76 remaining papers, 12 were manually chosen based on full-text analysis and included in this review paper (see Figure 3).

Table 3 provides the data we gathered from each study reviewed in this systematic review, focusing on general characteristics such as the year of publication, study setting, participant demographics, oncological diagnosis, inclusion and exclusion criteria, and cancer staging. Table 4 provides a detailed overview of saliva types, collection methods, centrifugation processes, storage conditions, laboratory analyses, and potential salivary metabolites associated with colorectal cancer or colorectal adenomas. Most studies use unstimulated whole saliva as the diagnostic material for analysis. Saliva was typically processed by centrifugation and stored at −80 °C until further analysis.

## 4. Discussion

### 4.1. Oral Microbiota

Bacterial biofilms are present in various anatomical sites throughout the human body [71]. The composition and abundance of biofilm-forming species are influenced by multiple factors, including age, diet, lifestyle habits, overall health status, the presence of foreign bodies (e.g., prosthetic heart valves), and personal hygiene practices [47,72]. The oral cavity alone is estimated to contain up to 700 distinct bacterial species, of which only approximately 280 have been successfully isolated, cultured, and taxonomically identified to date [73,74]. Further research is warranted, as numerous microorganisms remain uncharacterized and may play a pivotal role in the pathogenesis of malignant conditions within the gastrointestinal (GI) tract. Previous studies have indicated that the composition of the oral microbiota may serve as a predictive marker for gastrointestinal malignancies [69,75,76,77].

Recent research by Zhang et al., 2022 highlights the potential of *Fusobacterium nucleatum* as a biomarker for colorectal cancer, as they have found that the levels of salivary *F. nucleatum* were significantly higher in colorectal cancer patients’ saliva [69]. These Gram-negative anaerobic bacteria are predominantly found in the oral cavity and have been linked to various gastrointestinal malignancies, including colorectal cancer [77]. Genomic analysis of *Fusobacterium nucleatum* by Abed et al. demonstrated a high degree of genetic concordance between strains isolated from colorectal tumor tissues and those found in the saliva of colorectal cancer (CRC) patients, irrespective of their oral health status [78]. These results are consistent with findings from Komiya et al., who used random primer multiplex PCR to support the hypothesis that oral-origin *F. nucleatum* may translocate to and colonize colorectal tissues [79]. Furthermore, the markedly elevated levels of *F. nucleatum* in both saliva and fecal samples from CRC patients, relative to healthy controls, underscore its potential as a biomarker for non-invasive CRC diagnostics [78,79,80]. Other studies indicate that *F. nucleatum* can influence tumor progression through several mechanisms, such as promoting inflammation, enhancing cell proliferation, and inducing DNA damage in host cells [69,77,81]. *F. nucleatum* produces FadA (a unique adhesin required for binding and invading epithelial cells) and alters endothelial integrity through binding cadherins that may activate oncogenic pathways, such as Wnt/βcatenin, in colorectal carcinogenesis [77]. *F. nucleatum* also affects immune response through its Fap2 (Gal-GalNAc binding lectin) interaction with an immune receptor named TIGIT (T cell immunoreceptor with Ig and ITIM domains) present on T cells and natural killer cells [77,82]. This may inhibit immune cells activities against CRC [77]. Moreover, it has been shown that elevated levels of *F. nucleatum* correlate with poor prognoses and reduced overall survival rates in CRC patients [83]. These findings support the notion that *F. nucleatum* could serve as a valuable diagnostic and prognostic biomarker, aiding in early detection and risk assessment of CRC, thereby offering a promising avenue for improving clinical outcomes in gastrointestinal oncology [65].

In addition to *F. nucleatum*, several other bacterial species have been detected in the saliva as potential biomarkers for colorectal cancer (CRC). Notably, Rezasoltani et al., 2024 have discovered *Chloroflexi*, *Lactobacillaceae*, *Rivulariaceae*, *Calothrix parietina*, *Rothia dentocariosa*, and *Rothia mucilaginosa* microbes in the saliva of CRC positive patients, none of which were present in the saliva of healthy control group individuals [65,84]. In their studies, it was concluded that oral microbiota have the potential to differentiate individuals with colorectal cancer from healthy controls [65]. Uchino et al., 2012 in their study have discovered, through an oral examination, that the overall bacterial oral plaque accumulation was larger in patients with CRC than that in a group of healthy patients, and oral hygiene in the CRC group had decreased when compared with the healthy group, as they brushed less frequently [67]. In this study, the LEfSe (Linear discriminant analysis effect size) analysis showed that the numbers of *Pasteurella stomatis*, *Streptococcus anginosus*, *Solobacterium moorei*, and *Sphingomonas koreensis* were higher in patients with colorectal cancer when compared with the saliva of the control group [67]. All these microorganisms are normal microbiota of the oral cavity; however, their elevated numbers may be supplied to the large intestine from the oral cavity and they may take part in the carcinogenesis of CRC as the invasion of bacterial species producing genotoxins such as colibactin, promoting chronic inflammation, evading immune responses, and forming biofilms that alter the gut environment may induce an immune cell response that promotes tumor growth [67,85].

Conde-Pérez et al., 2024 have conducted a microbiome analysis on oral and intestinal samples from 93 colorectal cancer patients and 30 healthy controls (non-CRC) [60,86]. Studied patients’ oral status was recorded using Silness-Loe gingival index and DMFT. They have found that *Bacteroides fragilis* and *Parvimonas micra* have been implicated in CRC development, particularly in its enterotoxigenic form, which can induce inflammation and promote tumorigenesis through the production of toxins (e.g., BFT) [60,86]. These findings suggest that the oral and gut microbiomes play a significant role in CRC pathogenesis, indicating that a diverse range of bacterial species could serve as valuable biomarkers for early detection and prognosis of colorectal cancer.

### 4.2. Salivary microRNA

MicroRNAs (miRNAs) are short, non-coding RNA molecules approximately 22 nucleotides in length that modulate gene expression by promoting mRNA degradation and regulating protein synthesis [87]. In humans, miRNAs are frequently situated in genomic regions involved in the regulation of critical and sensitive cellular functions, including proliferation, apoptosis, angiogenesis, and cellular dysregulation, thereby contributing significantly to cancer development [88]. Altered miRNA expressions can interfere with the regulation of the cell cycle, cellular growth, and apoptosis [88]. Notably *miR-218* induces cell cycle arrest in colon cancer cells during the G2 phase [88]. This is achieved by inhibiting cyclin-dependent kinase 4 (CDK4) and elevating the p53 level, which serves as a transcription factor, activated in response to cellular stress, to inhibit cell proliferation and trigger cell death [88]. Pathology of the p53 pathway can contribute to the development of tumors [88]. Numerous studies have discovered an altered expression of miRNA in patients suffering from early stages of CRC, which may provide new ways for early detection and prevention of cancer development [89,90,91]. It has been proven that circulating miRNA can be isolated from whole saliva and is able to remain stable for the period of time required by clinical needs [92].

Rapado-González et al., 2019 identified five specific salivary miRNAs (miR-186-5p, miR-29a-3p, miR-29c-3p, miR-766-5p, and miR-491-5p) that demonstrated the ability to differentiate colorectal cancer (CRC) patients across stages I to IV from healthy individuals. Using a cohort comprising 51 CRC patients, 19 patients with adenomas, and 37 healthy controls, the miRNA panel achieved a sensitivity of 72.0%, a specificity of 66.67%, and an area under the curve (AUC) of 0.754 [64]. In a separate study, Koopaie et al., 2024 investigated salivary miRNA levels in 42 colorectal cancer (CRC) patients and 33 healthy individuals. They reported a statistically significant elevation in the salivary expression of miR-92a and miR-29a in CRC patients compared with healthy controls (*p* < 0.001). The diagnostic performance of miR-92a demonstrated a sensitivity of 95.24% and a specificity of 84.85%, while miR-29a showed a sensitivity of 95.20% and a specificity of 87.88%. Based on these findings, the authors concluded that salivary miR-92a and miR-29a levels may serve as reliable non-invasive biomarkers for the early detection of CRC [61]. Such high sensitivity and specificity numbers are very promising for the development of screening tests for CRC in the future, which may allow for earlier detection in early-stage patients.

Sazanov et al., 2016 conducted a study examining the expression of miRNA-21 in the saliva and serum of patients diagnosed with CRC [66]. Their findings revealed a statistically significant upregulation of miRNA-21 expression in both saliva and serum samples from stage II–IV CRC patients compared with healthy controls. Notably, the differential expression was more pronounced in saliva than in serum, suggesting that salivary miRNA-21 may serve as a more sensitive biomarker for CRC detection and monitoring.

### 4.3. Salivary Metabolites

Saliva is a biofluid that is produced by major and minor salivary glands and contains components derived from the mucosal surfaces, gingival crevices and tooth surfaces of the mouth [93]. Saliva is rich in various metabolomics in a similar way to the blood serum [94]. The oral cavity and saliva are places for many metabolic pathways that are prone to different ongoing pathologic conditions in various places across the body [95]. Although studying saliva metabolome profile shows many advantages towards blood, plasma, urine, and other samples, not enough research is yet performed [96].

Notably Bel’skaya et al., 2020 have conducted a study in which they have used a combination of saliva volatile organic compounds (VOCs) (acetaldehyde, acetone, propanol-2, ethanol, and methanol) to distinguish patients with colorectal cancer and gastric cancer from healthy controls [58]. Inclusion criteria for the patients were age between 30 and 70 years, absence of other diseases, no other treatments during the study, good oral health, and no consumption of alcohol two weeks prior to the study [92]. The colorectal cancer group consisted of 29 people (I (T2N0M0)—2, IIb (T4aN0M0)—3, IIc (T4bN0M0)—5, IIIc (T4bN1-2M0)—3, and IV (T4bN0-2M1)—5 people), the gastric cancer group consisted of 11 people (IIa (T3N0M0)—4, IIIa (T3N1M0)—2, IIIb (T3-4N2M0)—3, and IV (T1-4N0-2M1)—2 people) and the control group consisted of 11 healthy patients. The study succeeded in distinguishing with a sensitivity and specificity of 95.7 and 90.9% using acetaldehyde, acetone, propanol-2, and ethanol [58]. The authors have also been able to distinguish patients with CRC from patients with gastric cancer measuring previously mentioned VOCs while additionally measuring the methanol level; in this case, the sensitivity for detecting stomach and colorectal cancer was 80.0 and 92.3% [58]. Bel’skaya et al. have proven that it is crucial to perform further studies of saliva VOCs for the purpose of clinical oncology laboratory diagnosis, as they are promising in their diagnostic value.

Kuwabara et al., 2022 in their study have compared salivary metabolomics of CRC patients with patients with adenoma polyps present in the colon and healthy individuals [62]. The study included patients with colorectal cancer and patients with adenoma. Patients with other types of cancer, patients who underwent treatment, and patients with chronic diseases, such as diabetes, were excluded. A total of 2602 unstimulated saliva samples were collected from patients with CRC (*n* = 235), adenomas (*n* = 50), and healthy controls (*n* = 2317) [62]. Data were then randomly divided into training (*n* = 1301) and validation datasets (*n* = 1301) [62]. In their study, all of the studied metabolomics, including N-acetylputrescine, 4-methyl-2-oxopentanoate, and 5-oxoproline, were elevated in CRC; notably, lactate and pyruvate, the end products of glycolysis, were elevated only in colorectal cancer patients. They concluded that the Warburg effect (cancer cell transformation of glucose to lactate despite functioning mitochondria and oxygen availability) and glutaminolysis, which are linked to cancer metabolism, might be the cause of the observed characteristics in saliva [62,97]. The study successfully distinguished the groups with high sensitivity and specificity, showing the potential in future clinical use [62].

### 4.4. Protein and Enzyme Salivary Biomarkers

Bratei et al., 2023 in their series of studies were able to differentiate 28 patients confirmed with gastric cancer and 31 patients confirmed with colorectal cancer based on Maspin, MLH1, PMS2, and K-Ras biomarkers in saliva [59,98,99]. They have observed that the usage of MLH1 and PMS2 for differentiation between healthy patients and cancer patients was successful; the use of KRAS was for better and more conclusive differentiation between gastric cancer and colorectal cancer patients [59,98,99]. Waniczek et al., 2022 performed a study with a study group of 39 CRC patients and 40 control group patients (no history of cancer) over 60 years of age who underwent surgery in the general surgery department [68]. In their study, they have found that Chemerin, α-defensin 1, and TNF-α in saliva are effective in detecting early stages of CRC, with salivary concentrations of all analyzed cytokines showing 100% sensitivity and 100% specificity, showing to be suitable markers in the diagnosis of CRC, particularly in the initial diagnosis of CRC using tests which measure the concentration of proteins (antigens) in saliva [68]. Lázaro-Sánchez et al., 2019 have studied 20 patients with colorectal cancer and 10 healthy controls; their study discovered that salivary sHLA-G levels were significantly elevated in patients compared with the healthy control group [63]. Furthermore, patients with advanced-stage CRC (stages III–IV) had higher sHLA-G levels in their saliva than those in the early stages (I–II) [63]. They concluded that sHLA-G was a promising biomarker for diagnosing and predicting CRC prognosis, and a valuable molecular target for future research and development.

### 4.5. Salivary and Serum Oxidative Stress Biomarkers

Although there are no papers on salivary oxidative profile in patients suffering from colorectal cancer, there are papers regarding different gastrointestinal diseases, such as inflammatory bowel diseases, which can progress to CRC or indirectly cause the malignancy [100]. Szczeklik et al., 2018 has proven that salivary oxidative stress measured in saliva patients diagnosed with Crohn disease showed elevated malondialdehyde (MDA) levels and lowered catalase (CAT) levels when compared with the control group [101]. Their study suggested MDA could be further researched for future early diagnosis of IBD. Similarly, Rezaie et al., 2006 has found, in the saliva of patients diagnosed with Crohn disease, significant reductions in salivary levels of total antioxidant capacity (TAC), albumin, and uric acid (UA) levels and increases in nitric oxide levels and lipid peroxidation (LPO) concentrations [102]. Further studies regarding salivary oxidative stress in colon and rectal malignancies can possibly play a role in the prevention of colorectal cancer.

Zińczuk et al. have performed a study regarding oxidative biomarkers in serum in patients with colorectal cancer [103]. Their study included a study group of 50 patients (19 women and 31 men) and a control group of 40 healthy subjects. In their study, they have measured the levels of antioxidant barrier (Cu,Zn-superoxide dismutase (SOD), catalase (CAT), glutathione peroxidase (GPx), glutathione reductase (GR), UA, reduced glutathione (GSH)), redox status (TAC/total oxidant status (TOS), ferric reducing ability (FRAP)), and oxidative damage products (advanced glycation end products (AGEP), advanced oxidation protein products (AOPP), malondialdehyde (MDA)). The levels of UA, TOS, and OSI were significantly elevated, while the concentrations of AGE, AOPP, and MDA were also notably higher in CRC patients compared with healthy controls [103]. In contrast, the levels of GSH, TAC, and FRAP were markedly reduced in CRC patients [93]. They have concluded that redox biomarkers can serve as a potential diagnostic indicator of CRC advancement, although further studies are needed.

## 5. Conclusions

**Figure 4:**
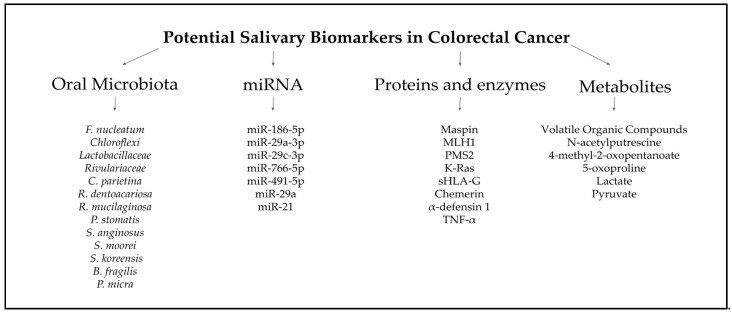
Potential salivary biomarkers in CRC [58,59,60,61,62,63,64,65,66,67,68,69].

The findings suggest that saliva provides a non-invasive, accessible, and reliable biofluid that can complement existing screening methods, such as colonoscopy and fecal tests, addressing their limitations in sensitivity, specificity, and patient compliance. A growing body of evidence, from elevated levels of oral *Fusobacterium nucleatum* and other dysbiotic bacterial species to highly discriminative microRNA signatures (e.g., miR-92a, miR-29a, miR-21), metabolic indicators of the Warburg effect (lactate, pyruvate, VOCs), and protein/oxidative markers (Maspin, Chemerin, sHLA-G, MDA), demonstrates saliva’s rich diagnostic potential. In conclusion, advancing the characterization and validation of salivary biomarkers for colorectal cancer promises to transform cancer screening paradigms (and potentially extend to the early detection of other malignancies) by delivering a noninvasive, cost-efficient, and highly patient-compliant diagnostic platform (Figure 4) [104]. Moving forward, largescale, multicenter studies integrating proteomic, transcriptomic, and metabolomic analyses are essential to optimize biomarker panels, rigorously evaluate their sensitivity and specificity, and establish clinically relevant reference thresholds. Such efforts will be critical to translating salivary diagnostics into precision oncology workflows, enabling individualized risk stratification, real-time treatment monitoring, and ultimately improving patient outcomes in the future.

## 6. Future Directions

SEER 21 data (2015–2021) show that 37% of cases present with spread to regional lymph nodes (“regional”) and 23% with distant metastases [105]. Similarly, European statistics report that roughly one in ten colorectal cancers is detected at a localized stage and about one in four at distant stage [106]. Saliva-based screening, by virtue of its noninvasive format, is expected to significantly enhance patient uptake compared with colonoscopy, thereby broadening population coverage, facilitating earlier detection of colorectal neoplasms, and markedly curbing total healthcare expenditures. Although the deployment of salivary assays may entail substantial initial costs (driven by the requirements for advanced analytical platforms, assay harmonization, and multi-omic integration), compared to the most commonly used for screening fecal occult blood test, as depicted in Table 5, these investments are ultimately offset by pronounced long-term savings derived from earlier-stage intervention and reduced therapeutic intensity (Table 6).

Moreover, integrating salivary biomarker research into clinical practice could also inspire novel therapeutic strategies. For example, targeting specific oral–gut translocated organisms like *Fusobacterium nucleatum* or biofilm-associated virulence factors may yield microbiome-modulating therapies (e.g., bacteriophage cocktails or quorum-sensing inhibitors) that disrupt tumor-promoting inflammation [112,113,114,115,116]. Likewise, salivary miRNA and metabolite panels that reflect dysregulated pathways, such as Wnt/β-catenin activation or the Warburg effect, could guide the development of tailored inhibitors or dietary interventions to normalize tumor metabolism [117]. Saliva-derived exosomes enriched in tumor- or stroma-specific signals may also serve as delivery vehicles for siRNA or immune-modulating agents directly to the tumor microenvironment [118]. Integrating salivary biomarkers with insights into the colorectal tumor microenvironment (TME) would also enable patient stratification and the development of novel therapeutics [119,120]. By leveraging noninvasive saliva profiling, clinicians could implement adaptive treatment regimens, switching or combining targeted therapies based on dynamic biomarker shifts, and accelerating the translation of microbiome- and metabolome-based interventions into precision oncology for colorectal cancer [121].

## 7. Limitations

Despite its promise, the field of salivary diagnostics faces several challenges. Most analyzed studies have been small, single-centered, and cross-sectional, limiting statistical power and precluding longitudinal assessment of prognostic kinetics. Pre-analytical variables, such as collection timing, patient fasting or oral hygiene status, and sample storage, are neither standardized nor universally reported, introducing variability and potential bias. Salivary composition is also influenced by many unrelated factors (e.g., periodontal disease, diet, and circadian rhythms), and few investigations have controlled for these confounders. Moreover, the practical aspects of implementation remain undefined: saliva collection may fall to dentists, nurses, or patients themselves, necessitating clear protocols and training. Another major disadvantage of salivary biomarkers is the lack of standardized reference values, which limits their clinical interpretation and comparability with well-established methods like colonoscopy and CT. Addressing these gaps through rigorously designed, multicenter prospective trials with standardized protocols will be critical to validating and translating salivary biomarkers into routine clinical practice.

## Figures and Tables

**Table 1:** Characteristics of blood, stool, and saliva collection.

	Blood Collection	Stool Collection	Saliva Collection (Spitting Method)
Invasiveness	High	Low	Low
Collection difficulty	Moderate (trained personnel)	Moderate (self-admin.)	Easy (self-admin.)
Biomarker range	High	High GI-specific	Moderate
Participant compliance	Moderate	Low–Moderate	High
Diagnostic versatility	High	Moderate (GI-focused)	Moderate
Sample stability	Stable	Variable	Variable
Reference Values	No	Yes	Yes

**Table 2:** Inclusion and exclusion criteria shown using the PECO framework.

Parameter	Inclusion Criteria	Exclusion Criteria
Population (P)	Patients of any age and gender	-
Exposure (E)	Colorectal cancer, colorectal adenoma	Other types of cancer
Comparison (C)	Comparison of saliva biomarkers to blood and stool biomarkers	-
Outcomes (O)	Salivary components as colorectal cancer biomarkers	-
Study Design	RCTs, case–control, cohort and cross-sectional studies published after 2014	Systematic reviews, case reports, conference reports, editorials, works not published in English, non-human studies

**Table 3:** General characteristics of included studies (CRC—Colorectal Cancer; CR—Colorectal; GC—Gastric Cancer; ND—No Data).

Author, Year	Setting	Study Group (F/M) Age	Control Group (F/M); Age	Diagnosis	Cancer Stages	Inclusion Criteria	Exclusion Criteria
Bel’skaya et al., 2020 [58]	Russia	*n* = 18 (CRC) 11 (GC) ND	*n* = 16 ND ND	CRC, GC	ND	(1) CRC or GC (2) age 30–70 years (3) good oral hygiene	(1) Other diseases (2) active treatment
Bratei et al., 2023 [59]	Romania	*n* = 31 ND ND	ND	CRC	ND	Randomly selected CRC patients	ND
Conde-Pérez et al., 2024 [60]	Spain	*n* = 93 CRC (36/57) 65, 70 (median)	*n* = 30 (23/7) 63, 64 (median)	CRC	ND	ND	(1) Other diseases (2) active treatment (3) genomic predisposition to develop CRC
Koopaie et al., 2024 [61]	Iran	*n* = 42 (19/23) 54.12 ± 13.98	*n* = 33 (18/15) 42.33 ± 12.50	CRC	I, II, III, IV	ND	(1) Other diseases (2) active treatment including blood transfusion in the last 3 years (3) pregnancy
Kuwabara et al., 2022 [62]	Japan	*n* = 235 (CRC) (105/130) 69.63 ± 12 *n* = 50 (CR Adenoma) (9/41) 61.81 ± 10.40	*n* = 2317 (1661/656) ND ND	CRC, CR adenoma	0, I, II(N1)/II(N2), Iva	Randomly selected CRC patients	(1) Other diseases (2) active treatment
Lázaro-Sánchez et al., 2019 [63]	Spain	*n* = 20 ND ND	*n* = 10 ND ND	CRC	I, II, III, IV	Patients with confirmed CRC	(1) Other diseases and 5 years prior (2) active treatment (3) pregnancy
Rapado-González et al., 2019 [64]	Spain	*n* = 51 (CRC) ND ND *n* = 19 (Adenomas) ND ND	*n* = 37 ND ND	CRC, CR Adenoma	ND	ND	(1) Other active diseases and 5 years prior (2) genomic predisposition to develop CRC
Rezasoltani et al., 2024 [65]	Iran	*n* = 40 (study group and control group) ND ND		CRC	0, I, II	(1) Patients experiencing bowel movement changes, rectal bleeding or abdominal pain (4) anemia (5) asymptomatic individuals aged 50 or above undergoing screening colonoscopy	(1) Other diseases (2) active treatment and antibiotic use 3 months prior to the study (3) a vegetarian diet (4) an invasive medical intervention within the past 3 months
Sazanov et al., 2016 [66]	Russia	*n* = 31 ND ND	*n* = 34 ND ND	CRC	II, III, IV	Patients with confirmed CRC	ND
Uchino et al., 2021 [67]	Japan	*n* = 52 ND 68.52 ± 10.6 years of age	*n* = 51 ND 54.49 ± 10.6 years of age	CRC	I, II, III, IV	Patients with confirmed CRC	(1) Other diseases (2) active treatment and antibiotic use 1 week prior to the study (3) constipation or diarrhea with Bristol Stool Form Scale scores ≤5 sampled (4) consumption of alcohol the previous day
Waniczek et al., 2022 [68]	Poland	*n* = 39 (21/18) 67.8 ± 10.3	*n* = 40 (21/19) 64.8 ± 9.4	CRC	I, II, III, IV	Patients with confirmed CRC	(1) Other diseases and 5 years prior (2) active treatment (3) substance abuse
Zhang et al., 2022 [69]	China	*n* = 207 (96/111) age < 63–102; > 63–105	*n* = 41 ND ND	CRC	I, II, III, IV	ND	(1) Incomplete medical records (2) no follow-up

**Table 4:** Detailed characteristics of included studies considering methods of collection and analysis of saliva (CRC—Colorectal Cancer; CR—Colorectal; GC—Gastric Cancer; ND—No Data).

Author, Year	Diagnosis	Type of Saliva and Method of Collection	Centrifugation (Time, RCF, Temperature) Storing	Analysis	Biomarkers
Bel’skaya et al., 2020 [58]	CRC, GC	(1) Unstimulated whole saliva (2 mL) (2) collection between 8 and 10 AM (3) fasting saliva collection (4) patients rinsed their mouth with water 10 min prior to sampling.	(1) 10 min, 10,000× *g*, ND (2) analysis immediately, no freezing	Chromatec-Crystal 5000 (Chromatec; Yoshkar-Ola, Russia)	Acetaldehyde, Acetone, Ethyl acetate, Methanol, 2-Propanol, 2-Buthanol, 1-Propanol, Ethanol, Catalase, Diene conjugates, Triene conjugates, Schiff bases, MDA
Bratei et al., 2023 [59]	CRC	ND	ND	PGSTAT 302 (Metrohm Autolab; Utrecht, Netherlands)	Maspin, MLH1, PMS2, and KRAS
Conde-Pérez et al., 2024 [60]	CRC	Unstimulated whole saliva ND	(1) 2 min, 4500× *g*, 4 °C (2) immediate resuspension in nuclease-free water and incubation for 1 h at 37 °C and 400 rpm in the presence of a specific enzymatic cocktail	MasterPureTM Complete DNA (LGC; Hoddesdon, UK) and RNA Purification Kit, AllPrepÒ DNA/RNA Mini kit (QIAGEN N.V.; Venlo, Netherlands), 1600 MiniG system (SPEX SamplePrep, LLC; Metuchen, NJ, USA)	Oral Microbiota
Koopaie et al., 2024 [61]	CRC	(1) Unstimulated whole saliva (2) collection between 8 and 10 AM (3) fasting saliva collection (4) dental and periodontal examinations	(1) ND (2) −80 °C	TRIzol reagent (Thermo Fisher Scientific; Waltham, MA, USA)	miR-92a and miR-29a levels
Kuwabara et al., 2022 [62]	CRC	(1) Unstimulated whole saliva (400 μL) (2) collection between 9 and 11 AM (3) fasting saliva collection	(1) no centrifugation, cloudy saliva was eliminated (2) −80 °C.	CE-MS (MasterHands; Keio University), LC–MS (Agilent MassHunter Qualitative Analysis and Quantitative Analysis software; Agilent Technologies, Inc.; Santa Clara, CA, USA)	N-acetylputrescine, 4-methyl-2-oxopentanoate, and 5-oxoproline, lactate, pyruvate
Lázaro-Sánchez et al., 2019 [63]	CRC	ND	(1) 3 min, 500×g, ND (2) −20 °C	ELISA kit sHLA-G (BioVendor; Brno, Czech Republic)	sHLA-G1 and HLA-G5
Rapado-González et al., 2019 [64]	CRC, CR adenoma	(1) Unstimulated whole saliva (5 mL) (2) collection between 9 and 10 AM (3) fasting saliva collection	(1) 15 min, 600× *g*, 4 °C (2) −80 °C	Nextera XT Index Kit (Illumina, Inc.; San Diego, CA, USA)	miR-186-5p, miR-29a-3p, miR-29c-3p, miR-766-5p, miR-491-5p
Rezasoltani et al., 2024 [65]	CRC	(1) Unstimulated whole saliva (400 μL) (2) collection between 8 and 12 AM (3) fasting saliva collection	(1) ND (2) −80 °C	QIAamp DNA Microbiome Kit (QIAGEN N.V.; Hilden, Germany)	Oral microbiota
Sazanov et al., 2016 [66]	CRC	(1) Unstimulated whole saliva (2 mL) (2) collection in the morning (3) fasting saliva collection (4) patients rinsed their mouth with water 10 min prior to sampling.	(1) 2 min, 12,000× *g*, ND (2) ND	TriReagent (MRC; Cincinnati, Ohio, USA) OT-1 (Synthol, Russia)	miR-21
Uchino et al., 2021 [67]	CRC	(1) Unstimulated whole saliva (2) collection upon waking up (3) fasting saliva collection (4) OMNIgene-ORAL OM-501 Saliva Microbiome DNA Collection Kit	(1) ND (2) analysis immediately, no freezing	GENE STAR PI-480 automated DNA (Kurabo Industries Ltd.; Neyagawa, Japan), QubitTM dsDNA HS (Thermo Fisher Scientific; Waltham, Massachusetts, USA), PCR (16S-27Fmod, 16S-338R)	Oral microbiota
Waniczek et al., 2022 [68]	CRC	(1) Stimulated whole saliva (2) no eating or drinking 20 min prior to saliva collection	(1) ND (2) −80 °C	LLC test (BioVendor; Brno, Czech Republic), Universal Microplate Spectrophotometer	Chemerin, α-defensin 1, and TNF-α
Zhang et al., 2022 [69]	CRC	(1) Stimulated whole saliva (2) mouth rinsing with water prior to the collection	(1) 2 min, 1000× *g*, ND (2) −80 °C	QIAamp DNA Mini Kit (QIAGEN N.V.; Hilden, Germany), TRIzol reagent (Thermo Fisher Scientific; Waltham, MA, USA), Qubit fluorometer (Thermo Fisher Scientific; Waltham, MA, USA), Bioanalyzer 2100 (Agilent Technologies, Inc., Santa Clara, CA, USA), AceQ qPCR Probe Master Mix (Vazyme Biotech Co., Ltd.; Nanjing, China), Bio-Rad CFX96 (Hercules, CA, USA), Roche Cobas e601 (Rotkreuz, Switzerland), Ribo-off rRNA Depletion Kit (Vazyme Biotech Co., Ltd.; Nanjing, China), VAHTS Universal V8 RNA-seq Library Prep Kit (Vazyme Biotech Co., Ltd.; Nanjing, China)	Oral microbiota

**Table 5:** Comparative cost analysis of saliva-based screening methods and traditional diagnostic methods for CRC in one patient [23,107,108,109].

Method	Estimated Cost per Patient (€)	What’s Included
Saliva—Oral microbiota (16S or Fn DNA)	150–250 €	Saliva kit, DNA extraction, qPCR or NGS sequencing, analysis report
Saliva—microRNA panel	100–200 €	Saliva kit, RNA extraction, qRT-PCR, report
Saliva—Proteins/enzymes	200–350 €	Saliva collection, multiplex ELISA or MS profiling, report
Saliva—Metabolites (LC-MS/SCFAs)	300–400 €	Saliva collection, metabolomics via GC-MS or LC-MS, bioinformatics, report
Colonoscopy (screening with possible biopsy/polypectomy)	~350–450 €	Procedure, sedation, biopsy/polyp removal, pathology
CT colonography (virtual colonoscopy)	400–600 €	CT imaging, radiologist interpretation, possible follow-up colonoscopy if positive
Capsule colonoscopy	600–900 €	Ingestible camera capsule, data retrieval, physician interpretation
Fecal immunochemical test (FIT) or gFOBT	~15–25 €	Sampling kit, lab analysis, result reporting

**Table 6:** Cost analysis of CRC treatment across disease stages I-IV per year [110,111].

Stage	Estimated Total Cost per Patient (€)	What is Included
Stage I	~8600 €	Initial hospitalization/surgery, follow-up care
Stage II	~12,700 €	Surgery, possible adjuvant chemotherapy, outpatient visits
Stage III	~13,000 €	Surgery with chemotherapy (adjuvant), follow-up
Stage IV	~23,000 €	Advanced care: hospitalisations, systemic therapy, palliative care

## Data Availability

No new data were created or analyzed in this study.

## Abbreviations

The following abbreviations are used in this manuscript:

CRC	Colorectal Cancer
FIT	Fecal Immunochemical Tests
gFOBT	Guaiac-based Fecal Occult Blood Tests
OBT	Occult Blood Tests
F	Female
M	Male
GC	Gastric Cancer
CR Adenoma	Colorectal Adenoma
IBD	Inflammatory Bowel Diseases
ND	No Data
SOD	Superoxide Dismutase
CAT	Catalase
GPx	Glutathione Peroxidase
GR	Glutathione Reductase
UA	Uric Acid
GSH	Reduced Glutathione
TAC	Total Antioxidant Capacity
TOS	Total Oxidant Status
LPO	Lipid Peroxidation
FRAP	Ferric Reducing Ability
AGEP	Advanced Glycation End Products
AOPP	Advanced Oxidation Protein Products
MDA	Malondialdehyde
OSI	Oxidative Stress Index
VOCs	Volatile Organic Compounds
